# Dutch translation and cross-cultural validation of the Adult Social Care Outcomes Toolkit (ASCOT)

**DOI:** 10.1186/s12955-015-0249-x

**Published:** 2015-05-13

**Authors:** Karen M van Leeuwen, Judith E Bosmans, Aaltje PD Jansen, Stacey E Rand, Ann-Marie Towers, Nick Smith, Kamilla Razik, Birgit Trukeschitz, Maurits W van Tulder, Henriette E van der Horst, Raymond W Ostelo

**Affiliations:** Department of General Practice & Elderly Care Medicine and EMGO+ Institute for Health and Care Research, VU University Medical Center, Amsterdam, The Netherlands; Department of Health Sciences and EMGO Institute for Health and Care Research, Faculty of Earth & Life Sciences, VU University, Amsterdam, The Netherlands; Personal Social Services Research Unit and Quality and Outcomes of Person-Centred Care Research Unit, University of Kent, Canterbury, Kent UK; Research Institute for Economics of Aging, WU Vienna University of Economics and Business, Vienna, Austria; Department of Epidemiology & Biostatistics and EMGO Institute for Health and Care Research, VU University Medical Center, Amsterdam, The Netherlands

**Keywords:** Translation, Cross-cultural validation, ASCOT, Social care, Quality of life

## Abstract

**Background:**

The Adult Social Care Outcomes Toolkit was developed to measure outcomes of social care in England. In this study, we translated the four level self-completion version (SCT-4) of the ASCOT for use in the Netherlands and performed a cross-cultural validation.

**Methods:**

The ASCOT SCT-4 was translated into Dutch following international guidelines, including two forward and back translations. The resulting version was pilot tested among frail older adults using think-aloud interviews. Furthermore, using a subsample of the Dutch ACT-study, we investigated test-retest reliability and construct validity and compared response distributions with data from a comparable English study.

**Results:**

The pilot tests showed that translated items were in general understood as intended, that most items were reliable, and that the response distributions of the Dutch translation and associations with other measures were comparable to the original English version. Based on the results of the pilot tests, some small modifications and a revision of the *Dignity* items were proposed for the final translation, which were approved by the ASCOT development team. The complete original English version and the final Dutch translation can be obtained after registration on the ASCOT website (http://www.pssru.ac.uk/ascot).

**Conclusions:**

This study provides preliminary evidence that the Dutch translation of the ASCOT is valid, reliable and comparable to the original English version. We recommend further research to confirm the validity of the modified Dutch ASCOT translation.

## Background

Social care services are provided to enable physically, mentally or emotionally impaired people to maintain independence and a good quality of life [1-3]. In the Netherlands, social care includes a range of domiciliary long-term care services, such as home care, transport and meals services, semi-institutional care, such as day care, and care in institutions, such as care homes and nursing homes. The ageing of the population will result in an increased need for social care in years to come [4]. Together with cutbacks on social spending [5], this means it becomes increasingly important to evaluate ‘value-for-money’ of social care services, in order to identify cost-effective alternatives and make the best use of limited resources [6].

To be able to estimate the value for money of social care services, it is important to evaluate outcomes of social care using instruments that reflect the character and objectives of social care. In (economic) evaluations of health services, outcome measures traditionally focus on the impact of the health service on health. Examples of such instruments are the EuroQol five-dimensional questionnaire (EQ-5D) [7] and the Short-Form Health Survey (SF-36) [8]. However, social care services aim to affect other domains than health alone such as independence, participation and overall well-being [3]. Therefore, there have been several initiatives in the last years to develop instruments that can be used to measure outcomes of care services that not directly aim to improve health. The Adult Social Care Outcomes Toolkit (ASCOT) [6] and the capability-based measure of general quality of life developed in the Investigating Choice Experiments for the Preferences of Older People programme (the ICEpop CAPability measure; ICECAP) [9,10] are the most well-known of these initiatives [11]. The ASCOT measures social-care related quality of life and was specifically developed in England for use in social care settings to reflect the impact and value of social care interventions.

Given the interest in the Netherlands to measure outcomes of social care services, the objective of this study was to translate the ASCOT into the Dutch language. The aim was to produce not only a Dutch version that is linguistically well translated, but also one that is culturally adapted to maintain the validity of the instrument at a conceptual level across the two countries [12]. This increases the confidence that the impact of social care services on quality of life is described in a similar manner in both countries. In this paper we describe the translation and cross-cultural validation process of the ASCOT for use in the Netherlands. We assessed the cross-cultural validity in pilot tests among frail older adults using think-aloud interviews, test-retest reliability, and a comparison of the response distributions and construct validity in England and the Netherlands.

## Methods

### Design and setting

In this study, the original English ASCOT was translated into the Dutch language according to the ‘translation and cross-cultural adaptation guidelines for self-report measures’ of Beaton et al. [12], including forward and back-translations. Pilot tests of the prefinal Dutch translation were done in a sample of community-dwelling frail older adults from the ‘frail older Adults: Care in Transition’ (ACT) study. The ACT study is a stepped wedge cluster randomised controlled trial designed to evaluate the (cost-) effectiveness of a geriatric care model for frail older adults living at home in two regions in the Netherlands and has been described in more detail elsewhere [13]. The prefinal version of the ASCOT was included in one of the follow-up measurements in one of the regions (190 respondents). Data were collected at the participant’s own home by means of computer assisted personal interviewing (CAPI). The response distributions obtained in the pilot tests of the Dutch translation of the ASCOT in the ACT study were compared to the response distributions using the original English version in a similar sample in England from the ‘Identifying the Impact of Adult Social Care’ (IIASC) study. The IIASC study is a survey of 990 publicly funded social care service users in England (Forder et al., in preparation). In this study, data collection either took place at the participant’s home by CAPI or was conducted by telephone.

Written or verbal informed consent was obtained from all study participants in the ACT study and the IIASC study. The ACT study received approval from the medical ethics committee of the VU University Medical Center (10/003). The IIASC study was approved by the Social Care Research Ethics Committee (12/IEC08/0049).

### ASCOT SCT-4

The Adult Social Care Outcomes Toolkit (ASCOT) was designed to capture information about an individual’s social care-related quality of life (SCRQoL) in eight domains: *control over daily life, personal cleanliness and comfort, food and drink, personal safety, social participation and involvement, occupation, accommodation cleanliness and comfort* and *dignity* [6]*.* Table 1 gives a description of domains. The toolkit includes a number of site-specific instruments for measuring SCRQoL [14]. For our purposes, we used the ASCOT SCT-4, a four-level self-report version to measure current SCRQoL in community settings. The ASCOT SCT-4 consists of 9 items, with each item representing one of the SCRQoL domains and two items representing the *Dignity* domain.

**Table 1:** **Domain descriptions and translation examples [©PSSRU at the University of Kent]**

**Domains**	**Description and translation of the items ‘Control over daily life’ and ‘Dignity filter question’**
**Control over daily life**	’The service user can choose what to do and when to do it, having control over his/her daily life and activities
	**Original version**
	1. Which of the following statements best describes how much control you have over your daily life?
	*By ‘control over daily life’ we mean having the choice to do things or have things done for you as you like and when you want.*
	- I have as much control over my daily life as I want
	- I have adequate control over my daily life
	- I have some control over my daily life but not enough
	- I have no control over my daily life
	**Final Dutch translation**
	1. Welke van de volgende uitspraken beschrijft het best in hoeverre u uw dagelijks leven zelf kunt inrichten?
	*Met het ‘zelf inrichten van uw dagelijks leven’ bedoelen we dat u de keuze heeft om dingen te doen of voor u te laten doen wanneer en hoe u dat wilt.*
	- Ik kan mijn leven inrichten zoals ik wil
	- Ik kan mijn leven voldoende zelf inrichten
	- Ik kan mijn leven in enige mate zelf inrichten, maar niet genoeg
	- Ik kan mijn leven niet zelf inrichten
**Personal cleanliness and comfort**	‘The service user feels he/she is personally clean and comfortable and looks presentable or, at best, is dressed and groomed in a way that reflects his/her personal preferences’
**Food and drink**	‘The service user feels he/she has a nutritious, varied and culturally appropriate diet with enough food and drink he/she enjoys at regular and timely intervals’
**Personal safety**	‘The service user feels safe and secure. This means being free from fear of abuse, falling or other physical harm’
**Social participation and involvement**	‘The service user is content with their social situation, where social situation is taken to mean the sustenance of meaningful relationships with friends, family and feeling involved or part of a community should this be important to the service user’.
**Occupation**	‘The service user is sufficiently occupied in a range of meaningful activities whether it be formal employment, unpaid work, caring for others or leisure activities’
**Accommodation cleanliness and comfort**	‘The service user feels their home environment, including all the rooms, is clean and comfortable’.
**Dignity**	‘The negative and positive psychological impact of support and care on the service users’ personal sense of significance’
	**Original version**
	*Dignity filter question*
	8. Which of these statements best describes how having help to do things makes you think and feel about yourself?
	- Having help makes me think and feel better about myself
	- Having help does not affect the way I think or feel about myself
	- Having help sometimes undermines the way I think and feel about myself
	- Having help completely undermines the way I think and feel about myself
	*ASCOT Dignity question*
	9. Which of these statements best describes how the way you are helped and treated makes you think and feel about yourself?
	- The way I’m helped and treated makes me think and feel better about myself
	- The way I’m helped and treated does not affect the way I think or feel about myself
	- The way I’m helped and treated sometimes undermines the way I think and feel about myself
	- The way I’m helped and treated completely undermines the way I think and feel about myself
	**Final Dutch translation**
	*Dignity filter question*
	8. Welke van de volgende uitspraken beschrijft het best hoe het hebben van hulp uw
	zelfbeeld beïnvloedt?
	- Het hebben van hulp heeft een positieve invloed op mijn zelfbeeld
	- Het hebben van hulp heeft geen invloed op mijn zelfbeeld
	- Het hebben van hulp heeft soms een negatieve invloed op mijn zelfbeeld
	- Het hebben van hulp heeft een volstrekt negatieve invloed op mijn zelfbeeld
	*ASCOT Dignity question*
	9. Welke van de volgende uitspraken beschrijft het best in hoeverre u zich gerespecteerd voelt door de manier waarop u wordt geholpen en behandeld?
	- Door de manier waarop ik word geholpen en behandeld voel ik me gerespecteerd
	- De manier waarop ik word geholpen en behandeld heeft geen invloed op hoe ik me voel
	- Door de manier waarop ik word geholpen en behandeld voel ik me soms niet gerespecteerd
	- Door de manier waarop ik word geholpen en behandeld voel ik me volstrekt niet gerespecteerd

The levels in each domain define the level of need: ideal state, no needs, some needs and high-level needs. The ASCOT SCT-4 adopts the capabilities and functioning approach [15,16] by distinguishing between capabilities and functionings in the response levels. Functionings are understood to reflect what a person is or does, whereas capabilities are understood as a person’s *ability* to function in a particular way, whether or not he or she chooses to do so. Both are considered valuable outcomes of social care [14]. The SCT-4 domains are phrased in the language of capabilities at the high quality of life end of the spectrum and in terms of functionings when reflecting low quality of life [14].

An overall SCRQoL index score can be calculated by applying English population preference weights, which reflect the relative importance of the different aspects of SCRQoL [6]. The first *Dignity* item (Dignity filter question) is not included in this score but is added to allow respondents to express how they feel about needing help. In development, it was found that this helped respondents answer the ASCOT Dignity question in the way it was intended, focusing on the impact of the way they are treated on their self-esteem. See Netten et al. for further information [17]. The index scale ranges from −0.171 (high needs on all domains) to 1, with ‘0’ equivalent to ‘being dead’ and ‘1’ being the ‘ideal’ social care-related quality of life state, where all needs are met to the desired level.

### Translation

The aim of the translation procedure was to reach conceptual equivalence between the original English version and the Dutch translation of the ASCOT, in order to maintain the content validity of the instrument at conceptual level across different cultures [12]. The ASCOT was translated into Dutch in 6 stages, as described below. As part of the translation procedure a cross-cultural validation was performed. The results from the four cross-cultural validation tests were used to refine the translation.

### Stage I: initial translation

The translation process started with an initial translation of the source version into Dutch. Reports describing the development of the ASCOT and the main guidance document were used as concept elaboration guides. A translation agency registered at the Netherlands Association of Interpreters and Translators (NTVG) and the European Association of Science Editors (EASE) was contacted to produce two forward translations into Dutch. The two bilingual translators from the agency had Dutch as native language. One of the translators had a medical background and was informed by the principal investigator (KvL) about the concepts and background of the ASCOT, the other translator was uninformed and had no medical background. The translators independently produced a translation of the item content, response options and instructions included in the ASCOT. A list of comments was added to the translation to highlight uncertainties or to provide other possible translations.

### Stage II: synthesis of the translations

The principal investigator and the translators discussed the comments and any discrepancies between the two translations and synthesized the results during a videoconference. Advice about unresolved issues and uncertainties were subsequently sought from a health scientist with nursing background (DJ), a Health Technology Assessment researcher (JB), and two older adults. As end result of this stage a synthesized version of the Dutch translation (first version) was put together by the principal investigator.

### Stage III: back translation

In order to assess whether the translated version reflected the same item content as the original version, two other bilingual translators produced back-translations into English, working from the first Dutch translation and blind to the original version. These translators were native English speakers. Again, one of the translators had a medical background and was informed about the concepts and background of the ASCOT, whereas the other was uninformed and had no medical background.

### Stage IV: expert committee

An expert committee, consisting of the Dutch authors of this paper, compared the back translations with the original version to review any discrepancies in meaning and suggested modifications to resolve the discrepancies. The back-translation and the suggested modifications of the expert committee to the first version of the Dutch translation were reviewed by the ASCOT development team, who provided feedback and some alternative modifications. This feedback was discussed within the expert committee after seeking additional advice from two bilingual health scientists. Eventually a prefinal version of the Dutch translation of the ASCOT was agreed on.

### Stage V: test of the prefinal version (cross-cultural validation)

The prefinal version of the Dutch translation was tested in a sample of frail older adults. These tests included an assessment of the content validity using ‘think-aloud interviews’ [18,19], an assessment of the test-retest reliability of the items and the total SCRQoL score, an assessment of the construct validity, and a comparison of the distributions of responses in a similar sample in England. The methods of these cross-cultural validation tests are described below.

### Stage VI: final version and appraisal of the adaptation process

Putting together all the evidence and experiences from previous stages and a German description of the ASCOT domains [20], a final meeting with the expert committee was organized in which a couple of outstanding issues were discussed and a slightly modified translation was produced. The modifications were proposed to ASCOT development team. Taking into account the feedback of the development team, the final version, including a new back translation, was submitted to the development team for appraisal of the adaptation process. This version was approved by the development team.

### Stage V: cross-cultural validation

The methods used in stage V of the translation procedure (test of the prefinal version) are described below. This stage concerned the cross-cultural validation of the prefinal version of the Dutch ASCOT, defined in the Consensus-based Standards for the selection of health Measurement Instruments (COSMIN) taxonomy as the degree to which the performance of the items on a translated instrument is an adequate reflection of the performance of the items of the original version of the instrument [21].

#### Content validity

The content validity, defined as the degree to which the content of an instrument is an adequate reflection of the constructs intended to be measured [21], of the prefinal version of the Dutch ASCOT was assessed using a think-aloud protocol in a qualitative study among 10 older adults living at home. These older adults were selected from the 3111 community-dwelling frail older adults who were previously approached for the ACT study, irrespective of their participation status. Interviews took place at the home of the respondents, where they completed the Dutch ASCOT while explaining their responses and opinions (think-aloud exercise). To assess whether the Dutch translation was accurate, explanations of responses were compared to the concept elaboration guide of the ASCOT.

#### Comparison with response distributions in England

The prefinal version of the Dutch ASCOT was administered in a sample of 190 frail older adults who participated in the ACT study. To assess whether the levels of need within the domains were interpreted similarly in the Netherlands as in England, the response distribution was compared to the response distribution of the original ASCOT in a similar sample from England. To accomplish this, the Dutch sample was matched with participants of the IIASC study. First, community dwelling respondents aged 65 years or older who received social services due to physical limitations or sensory impairment were selected from the IIASC study. Second, the vmatch module in Stata v13 was used to match the samples on sex, marital status, limitations in activities of daily living (all exact matches), self-perceived health (+/−1) and age (+/−2 years).

Chi-squared tests in Stata v13 were used to assess the relationship between item responses of the Dutch sample on the translated ASCOT and the item responses of the English sample on the original version.

#### Construct validity

The ACT subsample was used to compare construct validity of the Dutch ASCOT with the construct validity of the original English ASCOT, using the following definition of construct validity: the degree to which the scores of an instrument are consistent with hypotheses with regard to relationships to scores of other instruments or differences between relevant groups [21]. The construct validity of the original ASCOT was established in a previous study by Malley at al. [22] by testing hypothesised associations with other measures. We investigated whether the same associations were found using the prefinal version of the Dutch translation. To this purpose, we used variables that were available both in the ACT study and in the study of Malley et al.: marital status (married/not married), living situation (with others/alone), health-related quality of life (hr-QoL) measured with the three level EQ-5D-3L [7,23], a single item question about self-perceived quality of life (QoL) and limitations in a range of (instrumental) activities of daily living (ADLs): getting around outdoors, using the toilet, bathing, dressing, eating, paperwork/finances, grocery shopping, and preparing meals.

Malley et al. expected having a partner and living with others to be associated with lower levels of need on the two ASCOT items *Personal safety* (confirmed) and *Social participation and involvement* (not confirmed). Also, it was expected that lower levels of need on all ASCOT items were associated with higher self-perceived QoL and hr-QoL (confirmed). Lastly, it was expected that limitations in all ADLs were associated with higher needs on the *Control over daily life* item, limitations in personal care ADLs with higher needs on the *Personal cleanliness and comfort* item, and limitations in food-related ADLs with higher needs on the *Food and drink* item (all confirmed).

We hypothesized a-priori that the same associations would be found between the items in the Dutch translation of the ASCOT and other measures. To assess associations, we used one way analysis of variance (for hr-QoL) and chi-squared and Fischer exact tests for the other variables, in IBM SPSS Statistics 20.

#### Test-retest reliability

A retest measurement in the ACT subsample within 7–14 days after the first measurement was used to assess the test-retest reliability of the prefinal version of the Dutch ASCOT. Test-retest reliability is defined as the extent to which scores for patients who have not changed are the same for repeated measurements over time [21]. The retest measurement was performed using computer assisted personal interviewing as well. Test-retest reliability was estimated by calculating the quadratic weighted Kappa [24] for each item and the intraclass correlation coefficient (ICC_AGREEMENT_) [25] for the total index score. The ICC_AGREEMENT_ was calculated as the ratio of the between-subject variance and the total variance, based on the variance components obtained with the repeated measures analysis of variance (ANOVA) technique in IBM SPSS Statistics 20.

## Results

### Translation process and content validity

The two forward translations from Stage I showed only minor discrepancies. Because the principal investigator perceived some divergence from the content elaboration, additional advice was sought from health scientists and four older adults. This advice was incorporated in the version used for the back translations. The back translations revealed some additional deviations from the original English version. By reviewing the products and discussions from earlier stages, the expert committee produced and agreed on a prefinal version of the Dutch translation.

Six women and four men participated in the think-aloud interviews, aged between 75 and 100, and with varying levels of self-reported health and quality of life. The interviews showed that in general the items were understood as intended, although some issues were identified. These issues and the rationale for some of our translations decisions and modifications in Stage VI are described per item below. The domain description is shown in Table 1, as well as the original ‘*Control over daily life*’ and ‘*Dignity*’ items and the final Dutch translation of these items. The complete original English version and the final Dutch translation can be obtained after registration on the ASCOT website (http://www.pssru.ac.uk/ascot).

In several English items, the word adequate was used to denote the level in which needs were met. The literal translation for ‘adequate’ is a rather formal and ‘old-fashioned’ word in Dutch. We therefore chose to use the literal translation of ‘sufficient/enough’ (‘voldoende’).

#### Control over daily life

Respondents in the think-aloud exercise thought about several areas in which they could exert control, ranging from physical, mental, and financial control to bladder control. Some of the respondents mentioned they did not understand the meaning of this item. In order to change the annotation of the word ‘control’ with ‘being inspected’ in Dutch, and to avoid the focus on physical abilities, we decided in stage VI of the translation process to change the literal translation of ‘being in control’ to a more conceptual translation, which means literally as much as ‘being able to arrange your daily life’ (‘Je leven zelf kunnen inrichten’).

#### Personal cleanliness and comfort

We found a Dutch phrase for the phrase ‘I am able to present myself the way I like’ (‘Ik kan voor de dag komen zoals ik wil’) in the first response level of this item, that seemed to work quite well in the think-aloud exercise. Also, the translation of the word ‘presentable’ we used (‘toonbaar’) in the other response levels seemed to evoke the right reactions (this translation was extensively discussed in the review meetings); respondents thought about bathing, shaving, haircuts, make up and about making an effort to look their best.

#### Food and drink

Where the English word ‘get’ (in getting food and drink) is ambiguous about whether persons take care of food/drink himself or receives it, there is no such word in Dutch. We initially translate the phrase ‘getting food or drink’ to ‘having food or drink’ (‘eten en drinken hebben’), but changed it to ‘can eat and drink’ (‘kunnen eten en drinken’) in stage VI. The translation of ‘Having food and drink’ in Dutch was associated with the possession of food and drink, while ‘can eat and drink’ is in this context annotated with the ability of respondents to exert control over the process of eating and drinking.

#### Personal safety

Because respondents focussed on safety from crime while responding to this item, we changed the phrase ‘feeling safe’ (‘veilig voelen) to ‘feeling safe and secure’ (‘veilig en zeker voelen’) in all response levels in Stage VI.

#### Social participation and involvement

In Stage VI, we checked with the ASCOT development team whether the phrase ‘and I feel socially isolated’ in the last response level was considered as negative and atypical in English as in Dutch, because for some respondents this phrase sounded so bad they did not want to pick this answer, even though they felt lonely. The ASCOT team responded that the phrase was indeed meant to be severe. The comparison of response distributions showed that in both countries the percentage of respondents picking this response level was similar.

#### Occupation

Respondents focussed on their physical abilities to do things when explaining their answer on this item, which is based on the phrase ‘ability to do things the respondent values or enjoys with his/her time’. However, the domain description explains this domain as ‘to be sufficiently occupied in a range of meaningful activities’. In order to avoid the focus on ‘abilities’ we decided in Stage VI to leave the Dutch word for ‘to able’ (‘kunnen’) out of the translation. The back translation in the final version was: ‘I spend my time…’ (‘Ik besteed mijn tijd aan…’). Furthermore, some respondents expressed insecurity about the meaning of the translation we initially used for ‘… I value’ (‘…waar ik waarde aan hecht’). Therefore, we decided to change this in Stage VI to an alternative translation of ‘… I value’ (‘…die ik belangrijk vind’).

#### Accommodation cleanliness and comfort

While responding to this item, respondents focussed almost exclusively on the cleanliness of their accommodation. Therefore, we changed the word order in the final translation to ‘comfortable and clean’ as in Dutch the focus is on the first word.

#### Dignity filter question

Most respondents demonstrated a puzzled reaction to both *Dignity* items, as they did not understand how support and care would influence the way they think and feel about themselves. A typical reaction was “*I never think about myself*”. Maybe this reaction was evoked because the literal translation of ‘thinking and feeling about yourself’ is not used much in Dutch and was perceived as a deep philosophical reflection about yourself. However, respondents in the think aloud exercise did talk about how nice it was that they received support, even though it sometimes was difficult for them to adjust to the fact of being in need of help. The number of missing responses in the ACT study (both items were skipped by 10% of the respondents, whereas other items were skipped by 2% of the respondents at most) suggests as well that the translation of the *Dignity* items was difficult to answer. In the English sample the number of missing responses was smaller (6% and 3% for the *Dignity* filter question and the ASCOT Dignity question respectively). Therefore we altered the translation of the *Dignity* filter question in Stage VI to a phrase more common in Dutch ‘Having help … affects my self-image’ (‘Het hebben van hulp heeft … invloed op mijn zelfbeeld’).

#### ASCOT Dignity question

Again, although respondents indicated not to understand the translation of this item, they talked about all kind of examples in which they felt the way in which one was helped was experienced as negative or positive. However, respondents said they could not imagine how this would affect how they think and feel about themselves. Therefore, in Stage VI we changed the translation of ‘The way I’m helped and treated makes me think and feel better about myself’ to ‘The way I’m helped and treated makes me feel respected’ (‘Door de manier waarop ik word geholpen en behandeld voel ik me gerespecteerd’).

In conclusion, the first stages of the translation process did not result in many difficulties. The think-aloud interviews showed that in general the items were understood as intended. In response to some issues identified during these interviews, we made a small number of modifications by shifting the focus of an item or by using a less literal translation. The largest modification was made in the translation of the *Dignity* items, as most of the difficulties occurred in response to these items. All modifications were reviewed and approved by the ASCOT development team.

### Comparison with response distributions in England

The matching procedure with data from the subsample of the ACT study and IIASC study resulted in a match between 152 cases (Dutch sample) and 169 controls (English sample). Differences in characteristics of the matched respondents are shown in Table 2. Hr-QoL and self-perceived health was lower in the English controls. The difference in ADL limitations was statistically significant as well, but this difference was less than one limitation and therefore not considered clinically relevant.

**Table 2:** **Sample characteristics**

	**Subsample ACT study N = 190¶**	**Dutch cases (a subsample of ACT study matched to English controls) N = 169¶**	**English controls (IIASC study) N = 152¶**	**Difference between cases and controls**
				**P-value†**
Age. mean (SD)	82.39 (7.67)	82.04 (7.68)	80.68 (7.38)	0.11
Sex. % women	71.6%	78.1%	77.0%	0.81
Marital status. % not married (i.e. never married, widowed or divorced)	78.4%	80.5%	75.0%	0.24
Living situation. % alone	68.4%	72.8%	70.4%	0.64
EQ-5D (−0.594-1). Mean (SD)	0.59 (0.31)	0.59 (0.31)	0.37 (0.35)	<0.01
Utility-weighted ASCOT (−0.171-1). Mean (SD)^¶^	0.80 (0.16)	0.81 (0.16)	0.80 (0.16)	0.83
ADL limitations (0–8). Mean (SD)^ǂ^	2.14 (1.64)	2.16 (1.60)	2.70 (1.89)	<0.01
Self-rated health				
Excellent/very good. %	4.7%	4.7%	7.9%	0.05
Good. %	45.3%	45.0%	31.6%	
Fair. %	39.5%	40.2%	44.1%	
Poor. %	10.5%	10.1%	16.4%	

†Chi-square test (categorical) one-way ANOVA (continuous).

^¶^Missing Values: ACT (28), cases (20), controls (6).

^ǂ^Limitations that were available in the ACT study and the IIASC study: Bathing, dressing, using the toilet, eating, getting out of the house, grocery shopping, routine housework, paperwork/finances

+Missing values: ACT (2), cases (2), controls (1).

**Figure 1:**
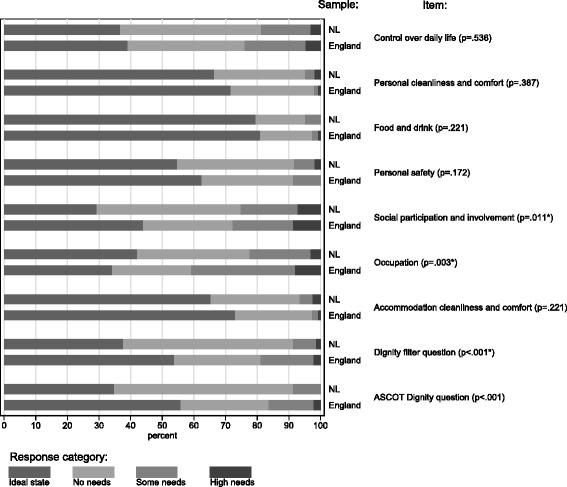
Response distributions ASCOT items in the Netherlands (NL) and England. Number of respondents NL = 169 (missing values between 0.6% and 10.7%); Number of respondents England = 152 (missing values between 0% and 5.9%)

Figure 1 shows the response distributions on the ASCOT items in both samples. Overall, the responses are distributed over the response levels in a similar pattern. Statistically significant differences in response distributions between the samples were found for the *Social participation and involvement, Occupation* and both *Dignity* items. Fewer respondents in the Netherlands indicated to have needs in the *Occupation* domain, which could be due to the focus in the Dutch translation of this item on being physically able to do things, or to the lower health status of the English controls. The think-aloud interviews also revealed some issues with the *Dignity* items, as described above. For the difference in respondents choosing the first level of *Social participation and involvement* we could not find an explanation in the think-aloud interviews.

### Construct validity

Table 3 shows whether the hypothesized associations between the ASCOT items and partner status, living situation, hr-Qol, self-perceived global Qol, and ADLs were statistically significant. Only associations for which an hypothesis was formulated by Malley et al. [22], were assessed. In contrast with the hypotheses, the *Food and drink* item was not statistically significantly associated with the food-related ADLs; *Personal safety* was not statistically significantly associated with marital status, living situation and hr-QoL; and *Social participation and involvement* not with marital status and living situation. Finally, *Dignity* was not statistically significantly associated with self-perceived QoL. However, the direction of the associations was as expected (Table 3).

**Table 3:** **Significance of associations between ASCOT items and other scales or items**

ASCOT item	**Control over daily life**					**Personal cleanliness and comfort**					**Food and drink**					**Personal safety**				
Response level	4 (high-level needs)	3 (some needs)	2 (no needs)	1 (ideal state)		4 (high-level needs)	3 (some needs)	2 (no needs)	1 (ideal state)		4 (high-level needs)	3 (some needs)	2 (no needs)	1 (ideal state)		4 (high-level needs)	3 (some needs)	2 (no needs)	1 (ideal state)	
N	N = 8	N = 27	N = 85	N = 67		N = 3	N = 5	N = 55	N = 125		N = 0	N = 11	N = 30	N = 146		N = 3	N = 11	N = 69	N = 106	
Partner status, % no partner																100%	64%	84%	76%	NS
Living alone, %																100%	64%	73%	66%	NS
Self-perceived QoL, % (very) good/excellent	14%	27%	57%	69%	***	0%	0%	38%	65%	***	-	18%	47%	60%	**	0%	9%	59%	59%	***
HR-QoL (EQ-5D), mean (sd)	0.18 (0.40)	0.35 (0.33)	0.63 (0.24)	0.70 (0.27)	***	0.48 (0.30)	0.06 (0.38)	0.55 (0.30)	0.64 (0.28)	***	-	0.27 (0.34)	0.61 (0.26)	0.61 (0.30)	***	0.42 (0.14)	0.49 (0.28)	0.60 (0.29)	0.61 (0.32)	NS
Getting around outdoors, %independent	0%	22%	47%	70%	***															
Using the toilet, %independent	75%	96%	99%	99%	**															
Bathing, %independent	25%	56%	78%	97%	***	33%	40%	75%	84%	**										
Dressing, %independent	38%	74%	87%	97%	***	100%	40%	84%	90%	**										
Eating, %independent	88%	96%	99%	100%	*						-	91%	100%	99%	NS					
Paperwork/finances, %independent	13%	59%	77%	91%	***															
Grocery shopping, %independent	0%	30%	51%	70%	***															
Preparing meals, %independent	14%	46%	64%	77%	***						-	40%	59%	61%	NS					
ASCOT item	**Social participation and involvement**					**Occupation**					**Accommodation cleanliness and comfort**					**Dignity**				
Response level	4 (high-level needs)	3 (some needs)	2 (no needs)	1 (ideal state)		4 (high-level needs)	3 (some needs)	2 (no needs)	1 (ideal state)		4 (high-level needs)	3 (some needs)	2 (no needs)	1 (ideal state)		4 (high-level needs)	3 (some needs)	2 (no needs)	1 (ideal state)	
N	N = 17	N = 34	N = 85	N = 52		N = 6	N = 40	N = 66	N = 75		N = 4	N = 11	N = 89	N = 62		N = 0	N = 15	N = 93	N = 57	
Partner status, % no partner	77%	71%	78%	85%	NS															
Living alone, %	53%	65%	67%	79%	NS															
Self-perceived QoL, % (very) good/excellent	19%	42%	64%	62%	***	0%	31%	58%	69%	***	0%	25%	43%	64%	***	-	33%	64%	54%	NS
HR-QoL (EQ-5D), mean (sd)	0.47 (0.41)	0.51 (0.30)	0.59 (0.30)	0.71 (0.25)	***	0.12 (0.39)	0.46 (0.28)	0.59 (0.30)	0.70 (0.25)	***	0.61 (0.25)	0.29 (0.41)	0.54 (0.30)	0.64 (0.29)	***	-	0.43 (0.35)	0.59 (0.31)	0.63 (0.27)	*

***significant at 1% level, **significant at 5% level, *significant at 10% level, NS not significant

Different from Malley et al. Empty cells indicate that was no hypothesis for the association between these measures, this association was not assessed.

Table 3 also shows whether the associations were in line with those reported by Malley et al. for the original English language version of the ASCOT. The non-significant associations with *Personal safety* may be due to the narrow focus on criminality of this translated item, as interpreted by the participants. Regarding the *Food and drink* item, this item included the phrase ‘I have food and drink’ in the prefinal version. This was changed in the final version to ‘I can eat and drink’, which will probably strengthen the association with the food-related ADLs. The think-aloud interviews and the comparison of response distributions already showed that the *Dignity* item was interpreted differently than intended. Difference in the statistical significance of associations could also be due to the smaller sample size in this study compared to Malley et al., and to small numbers in some of the cells in the cross tables of ascot items versus partner status and food-related ADLs .

### Test-retest reliability

After an average of 9 days, 147 older adults completed the prefinal version of the Dutch ASCOT for the second time. Kappa values for the individual ASCOT items ranged from 0.35 – 0.68 (Table 4), demonstrating slight to substantial reliability [26]. The lowest Kappa’s were estimated for the *Personal safety* and *Dignity* items. Cross-tabulations between the test and retest showed that respondents changed responses most from the ideal level to the no needs level and vice versa. The English preference weights show that the perceived differences between these levels are small compared to differences between other levels. Therefore, changes from the first to the second level and vice versa will not much affect the reliability of the total SCRQoL index score. The ICC for the total index score was good (0.71; 94% CI: 0.60-0.78).

**Table 4:** **Test-retest reliability of the Dutch translation of the ASCOT**

**Item**	**N**	**Quadratic weighted Kappa**	**Interpretation of Kappa [** 25 **]**
Control over daily life	147	0.66 (0.56 – 0.74)	Substantial
Personal cleanliness and comfort	147	0.68 (0.58 – 0.76)	Substantial
Food and drink	146	0.52 (0.39 – 0.63)	Moderate
Personal safety	147	0.44 (0.30 – 0.57)	Moderate
Social participation and involvement	147	0.66 (0.55 – 0.74)	Substantial
Occupation	144	0.60 (0.49 – 0.70)	Moderate
Accommodation cleanliness and comfort	146	0.67 (0.57 – 0.75)	Substantial
Dignity filter question	126	0.35 (0.18 – 0.49)	Slight
ASCOT Dignity question	125	0.44 (0.28 – 0.57)	Moderate

## Discussion

In this study we translated and validated the ASCOT SCT-4 into the Dutch language following international guidelines for cross-cultural adaptation [12]. Despite the rigorous translation procedure used to develop the prefinal version, the cross-cultural validation test revealed some discrepancies from the intended meaning and some validation issues. The *Safety* and *Food and drink* items were not as strongly associated with other measures as found in England and had moderate reliability. The think-aloud exercise suggested that the interpretation of these items focused on criminality issues and the availability of food. The results of all tests revealed that the *Dignity* items were poorly understood. Apart from these issues, the pilot tests showed that the translated items were in general understood as intended, that the reliability of the total index score of the Dutch translation was good and that the response distributions of the Dutch translation and associations with other measures were comparable to the original English version. The final version was developed to resolve these issues by some small modifications and a revision of the *Dignity* items, which was approved by the ASCOT development team.

Ideally, the tests should be repeated for the final version, but this was outside the scope of this study. However, apart from the *Dignity* items, only small modifications were made and we expect that the modifications will have a positive effect on the cross-cultural validation test.

A possible limitation of this study is that in the comparison of response distributions in a Dutch and English sample, matching was restricted to variables available in both datasets. For example, there was no information available in the ACT study about environmental characteristics and social contacts, which were previously found to be associated with the ASCOT [22,27]. Furthermore, although we matched the samples using five variables, we found some statistically significant and clinically relevant differences between the samples. Therefore, we cannot be sure whether differences between the response distributions were due to differences in sample characteristics or to the interpretation of items.

A subsequent step should be the development of Dutch preference weights, which represent the relative importance of the response levels of each domain for quality of life. Results from the think-aloud interviews and the comparison of response distributions suggests that there may be some differences between Dutch and English populations in the interpretation of the ‘severity’ of the response levels within the domains. The estimated preference weights will reveal these differences and by using country-specific preference weights differences in interpretation will be taken into account.

## Conclusions

In conclusion, we have successfully translated the ASCOT into the Dutch language, with the prefinal Dutch version demonstrating good measurement properties. The final Dutch translation can be obtained after registration on the ASCOT website (http://www.pssru.ac.uk/ascot). Research on the validity of the final version of the Dutch ASCOT as well as the responsiveness of the questionnaire to changes in quality of life as a result of social care service use in the Netherlands is warranted. As part of future work, the authors will focus on these questions and will conduct a study to elicit Dutch preference weights.

## References

[1] Inspectie voor de Gezondheidszorg, Zorgverzekeraars Nederland, LOC Zeggenschap in Zorg: *Kwaliteitsdocument 2013 Verpleging, Verzorging En Zorg Thuis*. 2013. https://www.zorgvoorkwaliteit.com/wpcontent/uploads/130805_Kwaliteitsdocument-VVT-2013.pdf. Access date: November 12, 2014.

[2] Department of Health (2006). Our Health, Our Care, Our Say: A New Direction for Community Services.

[3] Secretary of State for Health: *Caring for Our Future : Reforming Care and Support*. 2012.

[4] Lipszyc B, Sail E, Xavier A: *Long-Term Care: Need, Use and Expenditure in the EU-27*. 2012.

[5] Waldhausen A (2014). Care Services in Crisis?.

[6] Netten A, Burge P, Malley J, Potoglou D, Towers A-M, Brazier J (2012). Outcomes of social care for adults: developing a preference-weighted measure. Health Technol Assess.

[7] The EuroQol Group (1990). EuroQol–a new facility for the measurement of health-related quality of life. Health Policy.

[8] Ware JE, Sherbourne CD (1992). The MOS 36-item short-form health survey (SF-36). I. Conceptual framework and item selection. Med Care.

[9] Grewal I, Lewis J, Flynn T, Brown J, Bond J, Coast J (2006). Developing attributes for a generic quality of life measure for older people: preferences or capabilities?. SocSciMed.

[10] Coast J, Flynn TN, Natarajan L, Sproston K, Lewis J, Louviere JJ (2008). Valuing the ICECAP capability index for older people. Soc Sci Med.

[11] Makai P, Brouwer WBF, Koopmanschap MA, Stolk EA, Nieboer AP (2014). Quality of life instruments for economic evaluations in health and social care for older people: a systematic review. Soc Sci Med.

[12] Beaton DE, Bombardier C, Guillemin F, Ferraz MB (2000). Guidelines for the process of cross-cultural adaptation of self-report measures. Spine (Phila Pa 1976).

[13] Muntinga ME, Hoogendijk EO, Van Leeuwen KM, Van Hout HPJ, Twisk JWR, Van der Horst HE (2012). Implementing the chronic care model for frail older adults in the Netherlands: study protocol of ACT (frail older adults: care in transition). BMC Geriatr.

[14] Netten A, Beadle-Brown J, Caiels J, Forder J, Malley J, Smith N, Trukeshitz B, Towers AM, Welch E, Windle K: *Adult Social Care Outcomes Toolkit v2.1: Main Guidance. Volume PSSRU disc*. Canterbury: Personal Social Services Research Unit; 2011. [PSSRU discussion paper 2716/3]

[15] Sen A (1985). Commodities and Capabilities.

[16] Nussbaum M, Sen A (1993). The Quality of Life.

[17] Netten A, Ryan M, Smith P, Skatun D, Healey A, Knapp M (2002). *The Development of a Measure of Social Care Outcome for Older People. Volume PSSRU Disc*.

[18] Willis GB (2005). Cognitive Interviewing: A Tool for Improving Questionnaire Design.

[19] Collins D (2003). Pretesting survey instruments: an overview of cognitive methods. Qual Life Res.

[20] Trukeschitz B. Worauf es letztlich ankommt: Ergebnisqualität in der Langzeitpflege und -betreuung. *Kurswechsel* 2011;4:22–35.

[21] Mokkink LB, Terwee CB, Patrick DL, Alonso J, Stratford PW, Knol DL (2010). International consensus on taxonomy, terminology, and definitions of measurement properties for health-related patient-reported outcomes: results of the COSMIN study. J Clin Epidemiol.

[22] Malley JN, Towers A-M, Netten AP, Brazier JE, Forder JE, Flynn T (2012). An assessment of the construct validity of the ASCOT measure of social care-related quality of life with older people. Health Qual Life Outcomes.

[23] Brooks R (1996). EuroQol: the current state of play. Health Policy.

[24] Cohen J (1968). Weighted kappa: nominal scale agreement with provision for scaled disagreement or partial credit. Psychol Bull.

[25] De Vet HCW, Terwee CB, Knol DL, Bouter LM (2006). When to use agreement versus reliability measures. J Clin Epidemiol.

[26] Landis JR, Koch GG (1977). The measurement of observer agreement for categorical data. Biometrics.

[27] Forder J, Malley J, Towers A-M, Netten A (2014). Using cost-effectiveness estimates from survey data to guide commissioning: an application to home care. Health Econ.

